# Self-Reflection Protects Behavior from Volatile Beliefs Linked to Paranoia

**DOI:** 10.5334/cpsy.150

**Published:** 2026-02-18

**Authors:** Praveen Suthaharan, Santiago Castiello, Yuen-Siang Ang, Phil Corlett

**Affiliations:** 1 Interdepartmental Neuroscience Program, Yale School of Medicine, New Haven, CT, USA; 2 Department of Psychiatry, Yale University, New Haven, CT, USA; 3 Department of Social and Cognitive Computing, Institute of High Performance Computing, Agency for Science, Technology and Research, Singapore; 4 Wu Tsai Institute, Yale University, New Haven, CT, USA

**Keywords:** Uncertainty, belief volatility, metacognition, LLMs, prompt engineering

## Abstract

Processing uncertainty may be pathognomonic (characteristic of a disease) for some psychiatric conditions. Some people expect the world to change, even when it doesn’t. This tendency is central to paranoia, where individuals often anticipate threat or change without clear evidence. But what determines whether these beliefs translate into behavior? One possibility is that metacognitive structure – the coherence and depth with which one articulates their own thinking – acts as a buffer. An agent may endorse a belief but have sufficient accessory hypotheses to insulate it from action. To test this, we used metacognitive prompting in GPT-4 to score individual reflections on open-ended questions (e.g., *did you use any particular strategy?*) after completing a probabilistic reversal learning task. Individuals with higher paranoia demonstrate lower metacognitive structure (*t* = 5.98, *p* < 0.001), with metacognition attenuating the relationship between volatility belief and switching behavior (Δ = –15 pp, *p* < 0.001) even after controlling for reflection verbosity and general cognitive ability. These findings suggest that metacognition protects against uncertainty-driven instability, pointing to a key mechanism by which reflection protects against cognition under change. This work provides a novel framework to measure metacognition from behavioral task debrief questions.

## Introduction

Not all beliefs become behavior. Two people can hold similar beliefs – about themselves, others or the world – yet only one may act on them. Even within a single person, some beliefs spur action while others remain idle. This disconnect between what is believed and what is done is a longstanding challenge in both cognitive and clinical science (Bortolotti, 2015; Corlett, 2019; Evans, 2008; Frith, 2012). While belief content has been studied extensively, far less is known about what governs whether beliefs translate into actions.

Beliefs can take many forms, spanning social, emotional, and cognitive domains (Ford & Gross, 2019; Frith & Frith, 2012; Yon et al., 2020). In this work, we operationalize beliefs as the *latent estimates* that an individual forms about reward probabilities and environmental volatility. These estimates weight prediction errors driven by subsequent experiences, impacting updates to action values, thereby guiding decisions such as whether to stay or switch after a win. The link between beliefs and behavior is critical in psychiatry because beliefs often drive actions that shape mental health outcomes. For example, delusional beliefs can lead to avoidance, aggression or social withdrawal (Freeman, 2007; Freeman & Garety, 2014), while beliefs about hopelessness can drive inaction and disengagement in depression (Pizzagalli, 2014). Even in anxiety, exaggerated beliefs about threat can lead to excessive avoidance or compulsive checking (Rachman, 2002). These behavioral consequences are what clinicians aim to address through therapy or medication, yet the underlying mechanisms of how beliefs influence action are not fully understood.

One promising candidate that may influence this belief-to-action link is metacognition: the capacity to monitor, evaluate and structure one’s own thoughts (Flavell, 1979; Fleming & Dolan, 2012). Classic work distinguishes at least three canonical facets of metacognition (Fleming & Lau, 2014): *bias* (how confident someone tends to be overall), *sensitivity* (how well their confidence matches whether they are right or wrong), and *efficiency* (how well that match holds up, given the difficulty of the task). These facets are typically assessed in paradigms such as confidence calibration, post-decision wagering, or judgements-of-learning (JoL). More recent work highlights a fourth, behaviorally relevant facet – metacognitive *control*, the extent to which confidence guides future decisions, like switching strategies or seeking more information (Rouault et al., 2018). The present study focuses on a complementary fifth facet, metacognitive *structure*: the coherence and depth with which people articulate their own reasoning on reflection about their performance.

While bias, sensitivity, efficiency, and control each capture important aspects of metacognitive function, these constructs focus primarily on the accuracy or influence of metacognitive judgements rather than their internal organization. Metacognitive structure refers to the configuration of the metacognitive dimensions for one individual: how an individual comprehends the task (e.g., “I generally waited until I got –50 twice in a row before switching partners”), form and test judgements about emerging patterns (e.g., “At first I tried to count how many iterations a good card remained good”), evaluate their strategies and outcomes (e.g., “Switching did not work as well near the end. I had to stick with one even after a –50 because all others were worse”), decide on final rules or strategies (e.g., “I would usually stick with a student until failing an assignment, and then look for another student who performs better”), and express confidence or uncertainty in their reasoning (e.g., “I tried to take some risks but mostly stayed with my safer guesses”). These examples are illustrative of the qualities captured across the five dimensions of the metacognitive prompting framework (Wang & Zhao, 2024). These dimensions were not intended as one-to-one analogues of the metacognitive constructs, but as a practical rubric for characterizing the organization and coherence of reflective thought in open-ended text. This structural quality is distinct from whether a confidence judgement is correct or well-calibrated.

Metacognitive structure allows individuals to assess the reliability of their beliefs, inhibit impulsive responses, and maintain behavioral stability in the face of uncertainty (Allen et al., 2016; Rouault et al., 2018). Impairments in this capacity have been linked to poor insight, delusional ideation and risk for psychosis (Lysaker et al., 2014; Seow et al., 2021). Still, it remains unclear whether metacognition actively regulates belief-driven behavior, particularly in changing environments where beliefs are volatile.

Even in normative Bayesian learning schemes, beliefs can fluctuate when environments are volatile or evidence is noisy, which increases learning rate variability and can manifest as unstable choice patterns in reversal and change-point tasks (Behrens et al., 2007; Browning et al., 2015; Nassar et al., 2010; Reed et al., 2020). In such contexts, a structured reflective process may act as a stabilizing layer that insulates behavior from moment-to-moment belief noise until those beliefs are sufficiently integrated and organized to guide action. Rather than preventing adaptive learning, this buffering process may enhance it by ensuring that behavior reflects well-informed, internally coherent beliefs rather than transient fluctuations. Prior work has hinted at such higher-order properties, for example in the notion of metacognitive scaffolding that supports adaptive learning (Azevedo & Hadwin, 2005) and in evidence linking the quality of reflective insight to behavioral regulation (Desender et al., 2018; Rollwage et al., 2020; Rouault et al., 2019).

Paranoia has been associated with beliefs about task volatility. Individuals with high levels of persecutory ideation tend to switch strategies erratically in dynamic environments (Reed et al., 2020; Waltz & Gold, 2007) and to overestimate environmental changes (Cole et al., 2020; Powers et al., 2017). These patterns became especially apparent during the COVID-19 pandemic, when heightened uncertainty exaggerated both behavioral reactivity and belief volatility (Suthaharan et al., 2021). Yet, some individuals endorse conspiratorial ideas and yet tolerate volatility without becoming behaviorally erratic (Suthaharan & Corlett, 2023). This variation makes paranoia an ideal context for testing whether metacognition regulates the translation of belief into behavior.

First, we asked participants to complete a probabilistic reversal learning (PRL) task, which introduces shifting contingencies to probe how individuals adapt to change. Next, participants reflected on their experience by answering open-ended questions about their strategy and decision-making. We adopted a novel metacognitive prompting (MP) technique to evaluate these responses across five structured dimensions of reflective insight: comprehension, judgment, evaluation, decision and confidence (Wang & Zhao, 2024). Each response was scored using GPT-4, a large language model (LLM) instructed to assess the structure and quality of the participant’s reflection (Jenner et al., 2025). This allowed us to quantify metacognitive structure directly from natural language, and to examine how it relates to volatility beliefs and switching behavior across individuals.

We tested whether metacognitive structure influences the relationship between belief and behavior under uncertainty. First, we replicated that individuals with higher levels of paranoia do indeed exhibit both greater volatility expectations and increased switching behavior. We then predicted that these individuals would show lower metacognitive structure in their reflections. Critically, we hypothesized that metacognition would attenuate the belief–behavior link; when self-reflection was low, volatility beliefs would predict erratic switching; when self-reflection was high, this relationship would weaken. Together, these findings suggest that metacognitive structure may act as a stabilizing factor that can buffer against the behavioral consequences of uncertain or unstable beliefs.

## Methods

### Participants

A total of *N* = 486 individuals completed a computerized three-choice probabilistic reversal learning (PRL) task. All individuals were recruited through CloudResearch, an online platform that collects data from diverse and high-quality participants (Hartman et al., 2023). Data were collected in late 2020, when the platform’s bot-prevention and data-quality screening system (now branded as *Sentry*) was already in place. As in prior work (Suthaharan et al., 2021; Suthaharan & Corlett, 2023), we also manually vetted for (i) failed responses to a 3-item cognitive reflection check, (ii) finished the survey in < 5 min, or (iii) left compulsory questionnaire items blank.

The behavioral task data analyzed here were drawn from a previously collected dataset reported in earlier work (Suthaharan et al., 2021; Suthaharan & Corlett, 2023). Those prior reports focused on belief-updating behavior, whereas the present study introduces new analyses of the open-ended reflection responses, which had not been examined before. All participants were newly rescored and reanalyzed for the current hypotheses using the same inclusion criteria as in those previous work.

### Measuring Paranoia

Paranoia severity was assessed using the Revised Green Paranoid Thoughts Scale (R-GPTS) (Freeman et al., 2021), with a persecutory ideation cutoff (≥ 11) to split the data into high paranoia (*n* = 99) and low paranoia (*n* = 387). This scale has been used to distinguish clinically meaningful paranoia from normative variation.

### The Probabilistic Reversal Learning (PRL) Task

Participants completed one of two matched variants of a three-choice probabilistic reversal learning (PRL) task. One version with decks, and the other with avatars. Both versions had identical reinforcement structures and reversal criteria. Previous work confirmed no significant behavioral differences between the two iterations (Suthaharan et al., 2021); therefore, data from both task versions were pooled for analysis.

Each participant completed 160 trials. The task included both performance-independent reversals (occurring every 40 trials) and performance-dependent reversals (triggered after 9 of 10 consecutive selections of the best deck). A contingency *shift* occurred halfway through the task (at trial 81), altering the underlying reward probabilities from a 90-50-10 set to 80-40-20 set. These task features were designed to probe both reactive and anticipatory belief-updating under environmental volatility.

To quantify behavior, we focused on win-switching: the tendency to change choices after receiving a reward. This measure captures a form of decision instability that reflects how people react to positive outcomes. For each participant, win-switch rate was calculated as the proportion of trials in which they selected a new option after receiving a reward, relative to the total number of reward trials. This metric served as a proxy for behavioral flexibility and reactivity under uncertainty.

The focus on win-switch instead of lose-stay was grounded in consistent empirical evidence. Prior work using the same PRL paradigm has shown that neither empirical human data nor model-simulated data reveal any significant relationship between lose-stay behavior and paranoia (Reed et al., 2020). Whereas, win-switch behavior has consistently correlated with paranoid beliefs and volatility estimates, and this association has been repeatedly replicated across studies (Sheffield et al., 2022, 2023; Suthaharan et al., 2021, 2024). These findings indicate that win-switch behavior is a validated marker of belief volatility linked to paranoia, whereas lose-stay has not shown much meaningful associations in this context.

### The Hierarchical Gaussian Filter (HGF) Model

We modeled beliefs using the Hierarchical Gaussian Filter (HGF), a Bayesian model for perception, learning and action (Mathys et al., 2011). The model configuration used was based on prior work (Reed et al., 2020; Suthaharan et al., 2021; Suthaharan & Corlett, 2023). The HGF models three hierarchical levels (from top to bottom) of inference: belief about environmental volatility (level 3), belief about reward contingencies (level 2) and feedback (level 1). Of particular interest was the initial volatility prior (\[\mu _{3}^{0}\]), which reflects an individual’s anticipatory belief about the likelihood of environmental change. This parameter has previously been linked to delusional ideation and psychosis risk (Katthagen et al., 2022). We fit the three-level, mean-reverting HGF to each participant’s trial-by-trial data. The prior means and variances of the model parameters are detailed in Supplementary Table S1, where parameters with zero prior variance were fixed during model inversion. Choices were modeled using a volatility-dependent sigmoid (softmax) response model. This allows decisions to vary as a function of beliefs about environmental change. The HGF toolbox v.5.3.1 is freely available for download in the Translational Algorithms for Psychiatry-Advancing Science (TAPAS) package at https://translationalneuromodeling.github.io/tapas (Mathys et al., 2011, 2014). We installed and ran the package in MATLAB and Statistics Toolbox Release 2016a (MathWorks). The full perceptual model configuration file (*tapas_hgf_ar1_binary_mab_config.m*) used in our analyses is publicly available.

### Parameter Recovery

The three-level HGF used here has been rigorously validated for the same PRL paradigm. Previous studies demonstrated strong parameter recoverability and model identifiability for this variant (Cole et al., 2020; Reed et al., 2020; Suthaharan et al., 2024). We conducted parameter recovery using a custom simulation script (see GitHub) that reproduced the full agent-environment interaction within the PRL task (as in prior work (Suthaharan et al., 2024)). For each participant, we first fit the three-level mean-reverting HGF to their observed trial-by-trial choices, yielding individual-level perceptual (e.g., \[\mu _{3}^{0}\]) and response model parameter estimates. These participant-specific estimates were then treated as ‘ground-truth’ parameters for generating simulated behavior. For each participant, we simulated choices and outcomes iteratively over the entire PRL task structure, including the performance-independent and performance-dependent reversals and the mid-task contingency shift, thereby allowing the agent’s simulated experience to unfold exactly as it would for a real participant. On each trial, the agent’s choice was sampled from the volatility-dependent softmax using that participant’s true parameter values; the outcome (reward or no reward) was drawn from a Bernoulli distribution defined by the task’s reward schedule at that trial. This simulation was repeated 3 times per participant to generate multiple synthetic datasets that preserved both the individual’s estimated beliefs and the task’s non-stationary dynamics. Each synthetic dataset was then re-fit using the same HGF configuration, producing a set of recovered parameters. Recovery performance was quantified by correlating recovered parameters with the original fitted parameters across participants (see Supplementary Figure S9). Our present analysis builds directly on this validated framework to examine how individual differences in belief volatility interact with metacognitive structure.

### Model-Based Simulation Test of Metacognition Effect

We evaluate how changes in prior beliefs about environmental volatility (\[\mu _{3}^{0}\]) propagate to switching behavior in the generative model (HGF) and whether this propagation differs as a function of metacognitive structure. The same custom simulation script used for parameter recovery was applied, with participant-specific HGF parameters held fixed at their fitted values; only the initial prior on volatility \[\mu _{3}^{0}\]) was systematically varied. A grid of 15 \[\mu _{3}^{0}\] values was defined spanning the 15^th^ to 85^th^ percentiles of the empirical \[\mu _{3}^{0}\] distribution to remain within a realistic and numerically stable range. The full sequence of PRL choices-outcomes were simulated (as described in *Parameter Recovery*). The WSR was computed for each simulated {choice, outcome} sequence pair. These WSR values were averaged across 100 repetitions to obtain a subject-level expected WSR at each \[\mu _{3}^{0}\] value, yielding an individual \[\mu _{3}^{0}\] to WSR mapping for every participant. These subject-level mappings were then averaged across all participants to derive the model-predicted belief-behavior relationship (Supplementary Figure S7A). Participants were divided into low- and high-metacognitive groups based on ± 1 SD of the z-scored mean metacognitive score (Supplementary Figure S7B). Linear regression models were fit to the group-mean curves to estimate slope differences, and interaction model of the form \[WSR \sim \mu _{3}^{0} * MP\] was used to test whether the strength of the belief-behavior relationship differed between low- and high-MP participants. All simulation and analysis code is available in GitHub.

### Measuring Metacognition

We developed a custom scoring approach based on a large language model (LLM) prompted to emulate a metacognitive scientist. This approach uses the MP framework (Wang & Zhao, 2024), which defines the five core dimensions of metacognition: **Comprehension, Judgement, Evaluation, Final Decision**, and **Confidence** (see Supplementary Table S2 for details). These dimensions were selected as a structured and reproducible rubric for open-ended reflections, not as a direct one-to-one mapping onto the canonical metacognitive constructs (bias, sensitivity, efficiency, control and structure). The goal was to capture the reflective organization present in participants’ reasoning, using a practical scoring scheme that could be consistently applied by an LLM.

Each participant’s narrative was a combined response to two open-ended reflection prompts:

1. *Did you use any particular strategy or strategies? If yes, please describe*.
2. *Did you find yourself switching strategies over the course of the game?*

These were concatenated into a single string and input into the GPT-4 model (OpenAI et al., 2024), which was prompted to score the response on each MP dimension using a 5-point rubric (see Supplementary Table S3). Rather than returning a single discrete score, the model was asked to generate a probability distribution over all possible scores (e.g., [0.1, 0.2, 0.3, 0.2, 0.2]), allowing for uncertainty in borderline cases and greater scoring granularity.

To extract a continuous score per dimension *d*, we computed the expected value of the distribution:


\[{{M}_{d}} = \sum_{i=0}^{4} {{P}_{i}}* i\]

where *P_i_* is the probability assigned to score *i*. This resulted in five continuous metacognitive scores per participant, ranging from 0.0 to 4.0.

As a control analysis, reflection verbosity was also measured for each participant. Verbosity is defined as the number of characters in their combined written response (reflection length). We report this because LLM graders tend to assign higher quality scores to longer passages, a well-documented length bias (Zhao et al., 2024). This measure was computed using standard character count functions, and served as a proxy for verbosity (see Supplementary Figure S1). General cognitive ability was quantified using a composite score derived from two sources: participants’ self-reported education level (coded on a structured 1–8 ordinal scale from ‘less than high school’ to ‘doctoral or professional degree’) and their performance on three standard cognitive reflection test (CRT) questions (Suthaharan et al., 2021), a widely used proxy for reflective and analytic reasoning (Pennycook et al., 2016; Pennycook & Rand, 2019; Toplak et al., 2011). Each CRT question was scored as correct (1) or incorrect (0) using a rule-based scoring function that accepted numeric, text and unit-based variations of correct responses (e.g., ‘five’, ‘5 minutes’, ‘0.05’, etc.). A participant’s general cognition score was then calculated as the average of their education level and CRT accuracy.

### GPT-4 Access and Scoring Control

Participant reflections were evaluated programmatically through the OpenAI chat.completions.create() API, using the GPT-4-turbo model (see code on GitHub). A temperature setting of 0 was applied to ensure deterministic outputs, minimizing randomness across scoring iterations. This programmatic approach, combined with strict output formatting requirements and structured prompting (see Supplementary Figure S2), allowed reproducible, granular extraction of metacognitive scores. Explicit guidance encouraged the model to recognize subtle or abstract forms of insight (e.g., counterfactual reasoning, conditional logic, philosophical restraint). Reflections too short to meaningfully evaluate were auto-scored with a continuous value of 0.0 across all dimensions. For all other cases, both the model output and its accompanying rationale were saved for transparency (see data on GitHub).

### Human-model reliability

We benchmark the performance of GPT-4 scoring of metacognition against human raters. First, we randomly sampled a subset (~10%) of participant reflections (n ≈ 50; stratified to ensure balanced representation across high and low paranoia groups). Second, each reflection was independently scored by two raters, blind to GPT-4 outputs, using the same five-dimension rubric applied by the model. Third, we computed inter-rater reliability and assessed model-human agreement (see Supplementary Figure S3). Inter-rater reliability was quantified using a two-way random-effects intraclass correlation coefficient; ICC(2,1), which estimates the absolute agreement for single raters, and ICC(2,k), which estimates the reliability of the mean of *k* raters (Koo & Li, 2016).

### Moderation Analysis

We next tested whether metacognitive structure (\[\bar{M}\]) influences the relationship between initial prior on volatility beliefs (\[\mu _{3}^{0}\]) and switching behavior. Each participant’s average metacognitive score was computed by taking the mean across the five metacognitive dimensions:


\[\bar{M} = \frac{1}{5} \sum_{d=1}^{5} {{M}_{d}}\]

where *M_d_* denotes the continuous score for each dimension *d*. This gives us an index of overall reflective structure, ranging from 0.0 to 4.0. All regressors were z-scored prior to inclusion in the models to place them on the same scale, reduce potential multicollinearity in the interaction term, and make for easy interpretation. A binomial generalized linear model (GLM) logistic regression model was then formulated to predict win-switch rate (WSR) from initial prior on volatility, metacognitive structure, and their interaction:


\[WSR = {{\beta}_{0}} + {{\beta}_{1}}z\left(\mu _{3}^{0}\right) + {{\beta}_{2}}z\left(\bar{M}\right) + {{\beta}_{3}}\left(\mu _{3}^{0} * z\left(\bar{M}\right)\right) + \in \]

where *∈* represents residual error. The moderator (*β*_3_) tested whether the strength of the belief-behavior link depended on metacognitive capacity. To interpret the interaction, model-predicted probabilities of win-switching were examined at low (–1 SD) and high (+1 SD) levels of \[\bar{M}\], illustrating how belief-driven switching varies across metacognition. The moderation plot visualizes how volatility beliefs influence switching behavior at low and high levels of metacognitive structure, corresponding to individuals with relatively less or more self-reflective ability. Importantly, we include covariates controlling for verbosity and general cognition:


\[WSR = {{\beta}_{0}} + {{\beta}_{1}}z\left(\mu _{3}^{0}\right) + {{\beta}_{2}}z\left(\bar{M}\right) + {{\beta}_{3}}\left(\mu _{3}^{0}* z\left(\bar{M}\right)\right) + {{\beta}_{4}}z(V) + {{\beta}_{5}}z(C) + \in \]

where *z*(*V*) denotes standardized reflection verbosity and *z*(*C*) denotes standardized general cognitive ability. Model diagnostics supported the use of a binomial GLM over an ordinary least squares (OLS) model, as WSR was bounded between 0 and 1 and exhibited mean-variance coupling (see Supplementary Figure S4). The significance of the interaction was evaluated using a likelihood-ratio test (LRT) comparing nested models. We pre-specified a planned comparison, following recommendation for a priori contrasts (Granziol et al., 2025; Schad et al., 2020). Probability-scale contrasts (Figure 3B) were computed via nonparametric bootstrapping (1,000 iterations) to quantify changes in predicted switching across levels of metacognition. Contrast estimates were expressed in percentage-point (pp) units, representing absolute differences in predicted win-switch probabilities between conditions (e.g., a +7 pp differences indicates a 7% higher probability of switching).

### Statistical Analysis

#### General

All analyses were conducted using Python (version 3.11.9) with statsmodels, scikit-learn and matplotlib libraries. An alpha level of 0.05 and two-tailed tests were used throughout. Effect sizes were reported using Cohen’s d for group differences and unstandardized beta coefficients (*β*) for continuous regressions. Standard errors (SE), p-values and 95% confidence intervals were reported where appropriate.

#### Group Comparisons

Group-level differences in win-switch rate (WSR), initial prior on belief volatility (\[\mu _{3}^{0}\]), and metacognitive structure (\[\bar{M}\]) were evaluated using independent-samples t-tests or one-way ANOVAs (for factors with more than two levels, e.g., race). Cohen’s d was calculated for each contrast to estimate standardized effect sizes.

## Results

### Paranoia is associated with belief volatility and behavioral instability

**Figure 1:**
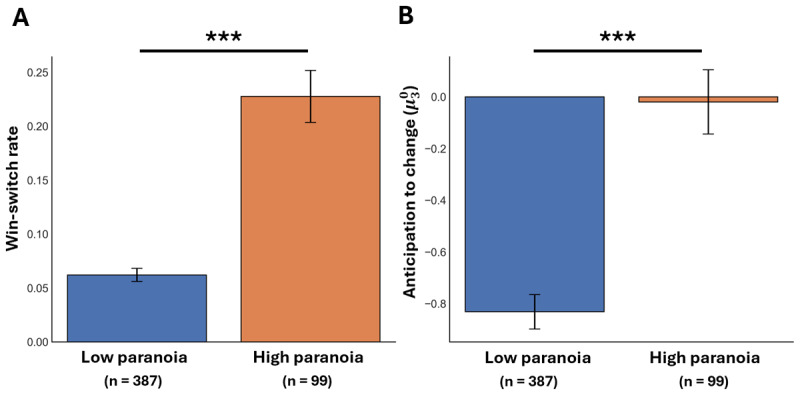
**Instability of behavior and volatility of beliefs are markers of paranoia**. We replicate prior work on linking behavioral markers from the probabilistic reversal learning task to paranoia. **(A)** Erratic win-switching behavior present in individuals who self-report having high levels of paranoia (*t* = –6.66, *p* < 0.001). **(B)** Elevated anticipation to volatility also present in high paranoia (*t* = –5.76, *p* < 0.001).

We first examined how paranoia relates to both behavioral and belief-level responses in the probabilistic reversal learning task. Participants were split into high and low paranoia groups based on the clinical cut-off for persecutory ideation (low: n = 387; high: n = 99). As predicted, individuals in the high-paranoia group exhibited significantly higher switching behavior in response to positive feedback (i.e., win-switching), a marker of behavioral instability (*t* = –6.66, *p* < 0.001, Cohen’s *d* = 1.09; Figure 1A). It was observed that participants who win-switched more often performed worse overall (*r* = –0.51, *p* < 0.001; Supplementary Figure S5). This inverse relationship indicates that excessive switching represents a maladaptive tendency that disrupts effective reward exploitation. This group also demonstrated elevated \[\mu _{3}^{0}\] values, reflecting elevated anticipation to volatility in the environment (*t* = –5.76, *p* < 0.001, Cohen’s *d* = 0.63; Figure 1B). Together, these findings replicate previous work showing that paranoia is associated with both volatile beliefs about environmental change and increased maladaptive switching behavior (Reed et al., 2020; Suthaharan et al., 2021).

### Metacognitive structure is reduced in individuals with paranoia

**Figure 2:**
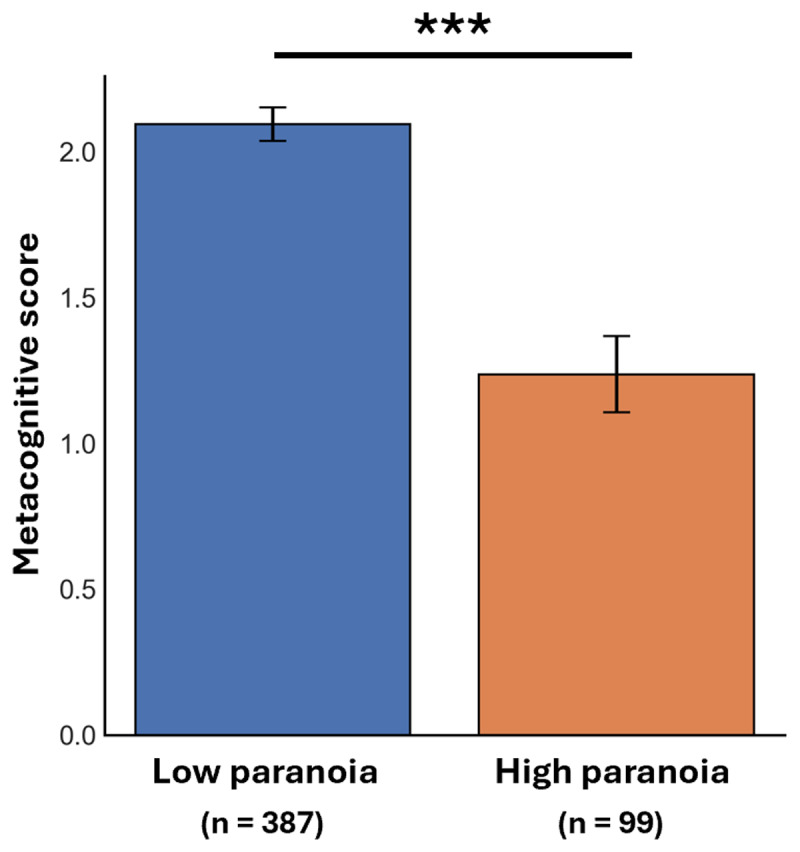
**Lower metacognition present in individuals who report having paranoia**. We compute a composite index of metacognitive structure by averaging across five structured dimensions: comprehension, judgment, evaluation, decision, and confidence. Individuals with higher levels of paranoia demonstrate significantly lower metacognitive structure (*t* = 5.98, *p* < 0.001), suggesting reduced ability to reflect on and structure their cognitive experience.

We next examined whether individuals with higher persecutory paranoia exhibited lower metacognitive structure. For each participant, an average metacognitive score was computed by aggregating across five dimensions—comprehension, judgment, evaluation, final decision, and confidence—scored via GPT-4 using a structured prompting technique (see *Methods*). As expected, the high-paranoia group demonstrated significantly lower metacognitive scores compared to the low-paranoia group (*t* = 5.98, *p* < 0.001, Cohen’s *d* = 0.73; Figure 2), a pattern that remained consistent when a stratified subset of reflections was scored independently by human raters and compared against GPT-4 scores (*r* = 0.81, *p* < 0.001; Supplementary Figure S3). This suggests that individuals who endorse paranoid beliefs reflect on their decision-making with less structure, coherence, or insight, aligning with prior work linking metacognitive deficits to psychosis-prone states (Lysaker et al., 2014; Rouault et al., 2018).

### Metacognition buffers the relationship between volatility beliefs and switching behavior

**Figure 3:**
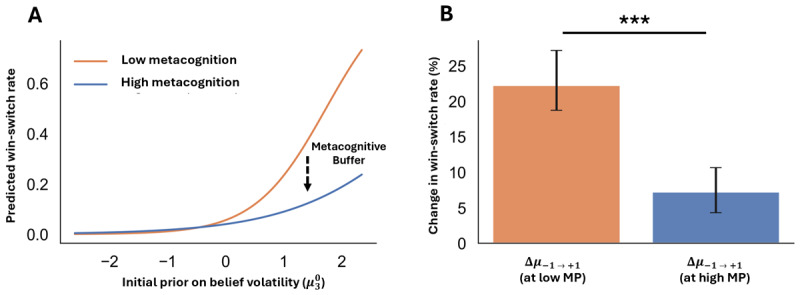
**Metacognition attenuates the link between belief volatility and behavior instability**. People who reflect more are less affected when beliefs become more volatile. **(A)** Predicted win-switch rate (WSR) from a binomial GLM fit: *wsr* ~ \[\mu _{3}^{0}\] * *MP* + *verbosity* + *cognition*. Curves show WSR across belief volatility at low self-reflection (MP = –1 SD; orange) and high self-reflection (MP = +1 SD; blue), accounting for controls. The widening gap at higher \[\mu _{3}^{0}\] illustrates a metacognitive buffer: increases in volatility translate to smaller increases in switching for participants who exhibit greater self-reflection. **(B)** Probability-scale contrast quantify that buffer. Bars show the absolute change in WSR (percentage points; pp) when \[\mu _{3}^{0}\] increases from –1 to +1 SD, evaluated at low and high MP; error bars are 1,000-bootstrap percentile 95% CIs.

We tested whether metacognitive structure influences the relationship between belief volatility and switching behavior. A binomial generalized linear model (GLM) was fit to predict changes in win-switch rate as a function of initial prior on volatility and metacognitive structure (see Methods). First, we motivate the reasoning for the specified model fit (see Supplementary Figure S4): the data are concentrated near 0 and the spread grows with the mean (min = 0.00, q1 = 0.00, median = 0.02, q3 = 0.099, max = 0.99; mean = 0.096, sd = 0.165; correlation of absolute residuals with fitted values = 0.452), and a Breusch-Pagan test rejects constant variance (*X*^2^ = 44.67, *p* < 0.001; F(5,480) = 9.72, *p* < 0.001). Second, we fit the GLM, controlling for reflection verbosity (measured as character count) and general cognitive ability (a composite of CRT and education level). Two stable patterns emerged (related to the main-effects): we observe higher volatility beliefs are linked to more switching (echoing prior work (Reed et al., 2020; Suthaharan et al., 2024)), and we observe higher self-reflection is linked to less switching (\[z\left(\mu _{3}^{0}\right)\]: OR = 3.64, 95% CI = [2.20, 6.04], *p* < 0.001, AME ≈ +0.098; *z*(*MP*): OR = 0.62, 95% CI = [0.43, 0.91], *p* = 0.015, AME ≈ –0.036). These results indicate volatility beliefs and self-reflection carry complementary information. Third, we assessed the influence of self-reflection on the link between beliefs and behavior (see Figure 3A-B). We asked the global question: *does self-reflection (MP) change the volatility belief (*\[\mu _{3}^{0}\]*) effect on win-switch rate?* We tested the MP * \[\mu _{3}^{0}\] interaction term and the interaction was insignificant (likelihood-ratio test; *X*^2^ (1) = 2.38, *p* = 0.123). However, we then asked the more practical question: *when beliefs about volatility increase, how much more do people with lower self-reflection win-switch than those with higher self-reflection?* This contrast shows that higher volatility beliefs raise win-switching much more when self-reflection is low than when it is high; +22 percentage points vs +7, which is a 15-point attenuation at high MP (Figure 3B, Δ = –0.15, 95% CI = [–0.134, –0.066], *p* < 0.001; GLM with controls, 1,000-bootstrap). This approach highlights that bounded variables bend near 0% and 100%, and thus a global test can miss real differences at meaningful points (i.e., low vs high MP). It could also be that the observed effects were partly driven by the covariates included in the model. We fit a covariates-only model predicting win-switch rate from reflection verbosity and general cognition. This model produced small average marginal effects (*AME*_*refl*_ = –0.0005, *AME*_*gcog*_ = 0.006) and did not explain substantial variance in win-switching (see Supplementary Tables S4–S5, Figure S6). Most importantly, this same attenuation emerged in an independent model-based simulation—with group differences in the \[\mu _{3}^{0} \to WSR\] slope formally tested using a linear regression interaction term (*p* = 0.005; Supplementary Figure S7)—indicating that metacognitive structure is associated with a reduced translation of volatility priors into switching behavior in the model. In the simulation, varying \[\mu _{3}^{0}\] alone was sufficient to induce changes in win-switch behavior, supporting a causal interpretation of the belief-behavior relationship rather than a purely correlational association. Together then, the results point to the idea that people higher in self-reflection are less pushed by volatile beliefs to erratically change their behavior after a win.

## Discussion

We find that individuals who exhibit stronger metacognitive structure are less likely to let volatile beliefs manifest as erratic behavior. We show that metacognitive structure attenuates the impact of belief volatility on behavioral instability. When individuals reflected less on their strategies, stronger expectations of environmental change led to more erratic switching. But with greater metacognition, those same beliefs no longer translated into reactive behavior. Erratic switching thus appears to follow from volatility overestimation, indicating that these two seemingly distinct symptoms are not merely comorbid but arise from a shared underlying computational mechanism. This suggests that metacognitive structure reflects more than self-awareness; it signals the presence of an internal model that allows individuals to simulate, evaluate and regulate their own beliefs before acting on them. Such a view aligns with recent accounts of psychiatric dysfunction as a disruption in these generative models of thought (Nour & Huys, 2023).

This finding extends and reframes existing work on paranoia. Previous studies have shown that individuals with high levels of persecutory ideation exhibit both heightened estimates of environmental volatility (Cole et al., 2020; Sheffield et al., 2022, 2023) and erratic decision-making in uncertain tasks (Reed et al., 2020). These features are often interpreted through the lens of aberrant learning or belief updating (Adams et al., 2013; Mathys et al., 2014). However, these accounts have rarely explained why unstable beliefs sometimes translate into unstable behavior – and other times do not. Our data suggest that this may depend on an individual’s capacity to reflect, structure, and evaluate their own thoughts. We speculate that paranoia may not be defined only by belief volatility, but by the failure to regulate those beliefs before they guide action.

This work builds on a growing body of literature positioning metacognition as a central control function in cognition and mental health. Metacognitive ability has been linked to insight in psychosis (Lysaker et al., 2014), functional outcome in schizophrenia (Pinkham, 2019), and resilience to delusional ideation (Rouault et al., 2018; Seow et al., 2021). Fleming and Frith proposed that metacognition serves a domain-general monitoring function across cognitive systems – shaping how beliefs, perceptions, and decisions are evaluated (Fleming & Frith, 2014). Our findings support this model and offer evidence that metacognitive structure not only reflects the quality of internal representations but gates their behavioral impact.

Existing tools for assessing metacognition often rely on tightly constrained paradigms or labor-intensive manual coding (Fleming & Lau, 2014; Güss, 2018). Validated self-report scales, while useful for trait-level beliefs about thinking, do not capture how participants actually describe and organize their reasoning to a specific task (Craig et al., 2020). Our approach leverages recent advances in natural language processing to measure metacognition in an interpretable and scalable way. While prior studies have used confidence calibration or post-decision wagering as proxies for metacognition (Desender et al., 2019; Fleming, 2024), we analyzed open-ended text responses and scored them along structured dimensions using GPT-4. This method allows for richer characterization of reflective thought – including coherence, evaluation, and decisional clarity (Wang & Zhao, 2024). It aligns with new work showing that large language models can identify self-reflection, theory-of-mind structure, and psychiatric symptomatology in free text (Hua et al., 2024; Kosinski, 2024). As LLMs grow more sensitive to human-like reasoning structures, so too will their ability to assess the quality of self-monitoring in real-world data.

In previous work, we showed that individuals who assume their social network shares their conspiracy beliefs experience less distress and behave more stably in uncertain environments (Suthaharan & Corlett, 2023). In that study we introduced the idea of a ‘*sacred canopy*’ – a structure of assumed shared belief within one’s social network – that can buffer against the psychological and behavioral consequences of volatility beliefs. People embedded in such canopies showed lower levels of anxiety, depression and paranoia, and expected less volatility during the same PRL task. Together with our current findings, it suggests that both internal (metacognitive structure) and external (social consensus) filters can protect against the translation of unstable beliefs into maladaptive behavior, converging on the same attenuation principle identified in our data. This has particular relevance for schizophrenia, where both metacognitive deficits and reduced social connectivity are well-documented. Individuals with schizophrenia often exhibit diminished metacognitive capacity (Pinkham, 2019) and maintain smaller, sparser social networks (Degnan et al., 2018), potentially lacking both protective filters. Future research should examine whether bolstering either – or ideally both – can disrupt the volatility-to-behavior pathway in clinical populations vulnerable to belief-driven instability.

We also observed that individuals with high paranoia tended to produce brief self-reflections on their task strategy (Supplementary Figure S1). While reflection length is not a direct measure of metacognitive ability, such reduced elaboration may reflect broader difficulties in expressing or articulating one’s own cognitive processes. Previous work has shown that individuals with schizophrenia often exhibit impoverished verbal output and reduced thematic elaboration (Goldberg, 2023; Lysaker et al., 2005). These expressive constraints may relate to more fundamental impairments in metacognitive access: the capacity to monitor, represent and report one’s own thinking. In this light, the brevity of responses in high paranoia individuals may not simply reflect task disengagement but may signal disruptions in reflective expressiveness that parallel broader metacognitive dysfunction.

Brief reflections may capture ancillary cognitive skills (e.g., recall, narrative organization, expressive fluency) in addition to metacognition. These processes could influence how coherently participants describe their strategies, even if their underlying metacognition constructs are unchanged. The capacity to remember task sequences or to verbally structure one’s reasoning might therefore contribute to higher MP scores, reflecting the organization of thought rather than metacognition alone. This does not diminish the relevance of these reflections but rather broadens interpretation; the ability to represent one’s own reasoning in language may itself be an important facet of reflective cognition. The close correspondence between GPT-4 and human ratings indicates that the metacognitive index reflects stable properties of reflective organization rather than artifacts of automated scoring. This suggests that GPT-4 is not generating arbitrary structure but capturing meaningful variation in metacognitive structure.

At a theoretical level, our findings build on and extend existing models of belief-updating. Bayesian learning frameworks, such as the Hierarchical Gaussian Filter (Mathys et al., 2014), often model behavior as a function of inferred volatility and learning rate. These models typically treat belief as the proximal driver of action, but emerging work suggests that behavior also depends on higher-order control processes that regulate how such beliefs are expressed (Fleming & Daw, 2017; Friston et al., 2015; Pezzulo et al., 2018). Our data are consistent with a reflective filter that determines whether internal beliefs are expressed behaviorally. This perspective resonates with normative accounts of meta-control, where policy precision and decision thresholds are modulated by beliefs about the reliability of one’s own inferences (Bénon et al., 2024; Shenhav et al., 2017). The importance of such reflective control is echoed beyond human cognition – recent work has shown that large language models, through large-scale pre-training, also develop emergent capabilities for self-correction and structured reflection (AI et al., 2025). It highlights that reflection may be a general mechanism of stabilizing behavior in the face of noisy internal signals. This opens a space for formal models of belief-to-action gating within existing generative frameworks, where metacognition operates not just as a monitor, but as a decision threshold. Notably, the fact that the metacognition effect is visible in the generative simulations suggests that it is at least partly expressed through parameters already captured by the fitted hierarchical model, rather than emerging solely as an ‘extra-model’ deviation from predicted behavior. One possibility is that metacognition covaries with how strongly inferred volatility propagates down the hierarchy (e.g., via coupling parameters such as *κ* that determine how much ‘the world is changing’ beliefs modulate how quickly I update reward beliefs), or with downstream decision parameters that translate beliefs into switching. An open question is whether a separable component of metacognitive regulation remains after accounting for these fitted mechanisms—i.e., whether individuals with higher metacognition show smaller empirical deviations from the model-predicted \[\mu _{3}^{0} \to WSR\] mapping even when volatility priors are matched. Distinguishing parameter covariation from an additional metacognitive gating process will require models that integrate trial-level behavior with momentary reflective reports (i.e., that is knowing what the person was thinking at that exact moment, not just what they chose), or extensions in which metacognition explicitly modulates belief-to-action expression. These findings raise the possibility that core features of schizophrenia – such as delusions or disorganized behavior – may not arise solely from abnormalities in belief formation or updating, but also from impairments in the generative models that support self-monitoring and behavioral gating. If metacognition functions as a reflective control layer that filters which beliefs are enacted, then deficits in this layer could explain why certain individual with unstable beliefs behave erratically while others do not. In this view, schizophrenia may involve not only aberrant priors about the world (e.g., heightened volatility) but also a weakened capacity to internally simulate, evaluate and regulate those beliefs before they shape action. Future work could formalize this interaction by incorporating metacognitive gating into computational models of psychosis, offering a richer framework that spans from low-level prediction errors to high-level failures of self-reflective control.

There are also translational implications. Cognitive behavioral therapy (CBT) and related interventions explicitly target how patients reflect on and appraise their beliefs, whereas much of psychiatry – particularly within its diagnostic and biological traditions (Kotov et al., 2017; Regier et al., 2013) – has historically emphasized what patients believe. Two individuals may hold similar suspicions or expectations, yet only one will act on them in destabilizing ways. Metacognitive structure may explain that difference. Interventions such as metacognitive training (Moritz et al., 2014), insight therapy (Musket et al., 2024), and guided reflection exercises (Zulfikar et al., 2025) may not need to alter belief content directly to reduce behavioral dysfunction. Instead, they may work by restoring the capacity to recognize and restrain unstructured thinking (Moritz et al., 2023).

## Limitations

Still, there are limitations. Our measure of metacognition relied on brief reflections and language model scoring. While recent work supports the reliability of LLM-based annotation (Gilardi et al., 2023; Tan et al., 2024), this field is still emerging. We did benchmark for model reliability of our scoring approach, though its scope was limited to a single rubric and a subset of reflections. Broader validation across tasks, populations, and languages will be critical. Moreover, recent studies show that GPT-4 can encode demographic biases in evaluation tasks by systematically favoring certain races, ethnicities or genders (Zack et al., 2024). In line with these concerns, we assessed whether our LLM model exhibited demographic variation (see Supplementary Table S6 for demographic distribution). We observed no evidence that the scoring process disadvantages on gender (*t*_422_ = 0.9, *p* = 0.368). We did however see differences across race (F_3,55_ = 7.87, *p* < 0.001), but this pattern was not unique to GPT-4. It was also present in the two independent human raters (rater 1: F_2,7.35_ = 3.63, *p* = 0.08; rater 2: F_2,7.73_ = 7.69, *p* = 0.015; human raters (averaged): F_2,7.42_ = 5.29, *p* = 0.037) and so may reflect sampling differences rather than model bias. To further contextualize this pattern, we examined reflection characteristics across racial groups (see Supplementary Figure S8) and found significant differences in reflection length (Supplementary Figure S8A; F_3,60.39_ = 5.38, *p* = 0.002) and paranoia scores (Supplementary Figure S8B; F_3,54.26_ = 4.49, *p* = 0.007). These factors provide a plausible explanation for the observed demographic variation as these covariates are associated with MP scores. At present, though, our preliminary analyses suggest that any apparent race-related differences in scoring are not necessarily indicative of bias intrinsic to the model itself. An important direction for future work will be to understand how lived experience, race, and response structure interact in LLM-based scoring. Fairness evaluation remains a priority for follow-up validation.

Our design was also cross-sectional and observational; therefore, while we can say metacognition influences the link between belief and behavior, we cannot say increasing metacognition will reduce behavioral instability. We avoid making any such causal claims about real-world changes in metacognition or behavior beyond the model-based simulations. And while we focused on persecutory paranoia, the underlying mechanism may generalize to other experiences marked by belief volatility – such as obsessive doubt, ruminative anxiety, or epistemic mistrust.

Future work should explore whether this reflective buffering operates dynamically over time, changes with stress or fatigue, and can be enhanced through intervention. It may also be possible to develop hybrid models that integrate trial-level behavior with momentary text reflections to predict state-level fluctuations in metacognitive regulation.

## Conclusion

We identified metacognition as a key buffer between belief and behavior using a novel, scalable approach to score natural reflections with artificial intelligence. This work sets the stage for new ways to detect, understand and ultimately intervene on cognitive volatility and instability in clinical and everyday settings.

## Data Accessibility Statement

The data and analysis scripts can be found at https://github.com/psuthaharan/metacognition-prl.

## Additional File

The additional file for this article can be found as follows:

10.5334/cpsy.150.s1 Supplementary Material. Supplementary Figures S1–S9 and Supplementary Tables S1–S6.
