# Supplementary material for: Self-Reflection Protects Behavior from Volatile Beliefs Linked to Paranoia
Source: Comput Psychiatr. 2026 Feb 18;10(1):18–34. doi: 10.5334/cpsy.150 (PMC12922661; doi:10.5334/cpsy.150)
Supplement: Supplementary Material. — Supplementary Figures S1–S9 and Supplementary Tables S1–S6. [file cpsy-10-1-150-s1.pdf]

FIGURES

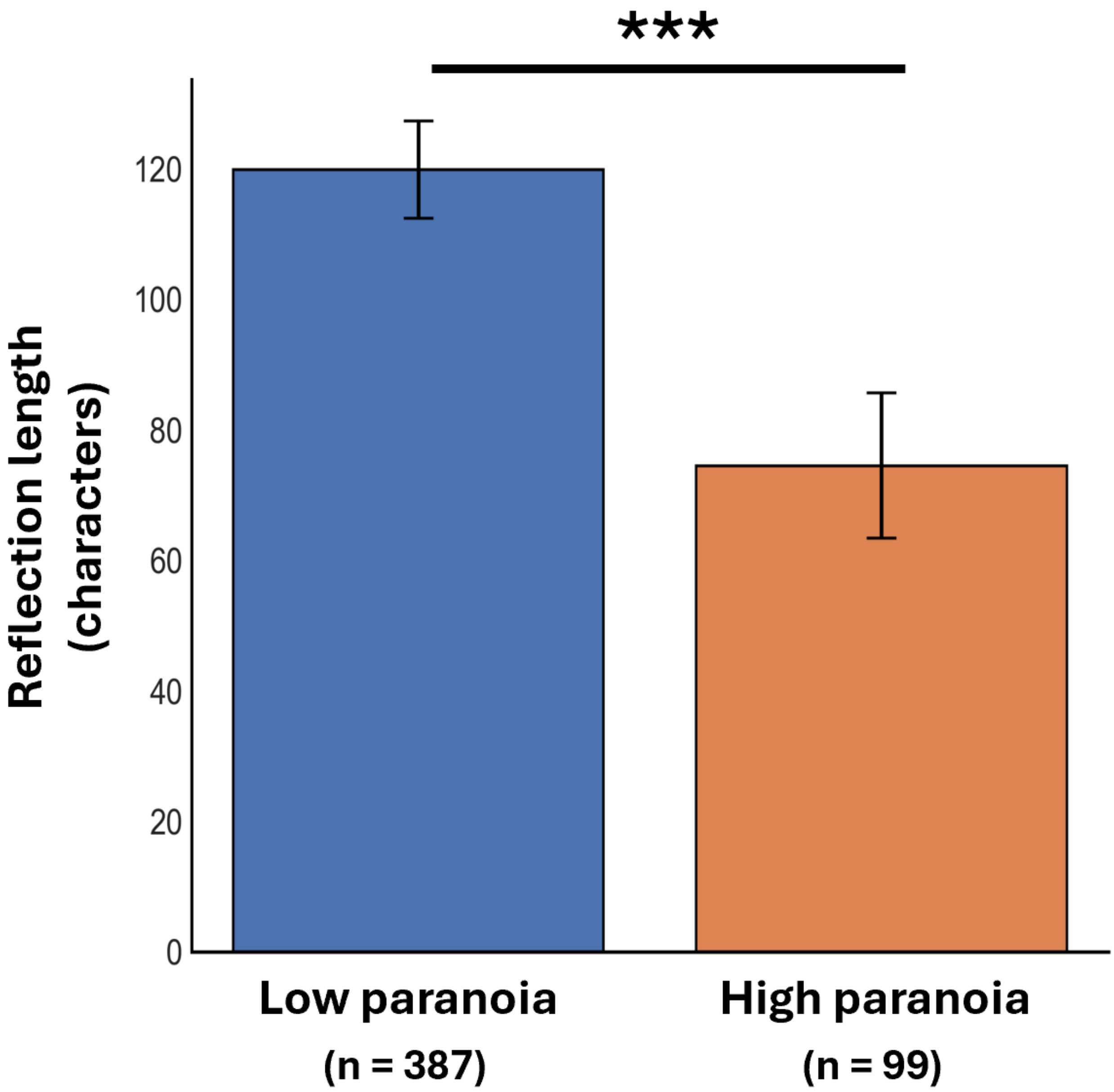

Supplementary Figure S1. Reflection length by paranoia group. *A bar plot showing the average character count of combined reflection responses by paranoia group. Individuals with elevated paranoia wrote significantly shorter responses (t = 3.39, p < 0.001, Cohen's d = 0.32).*

Prompt: You are a metacognitive scientist evaluating a participant's reflection on a three-choice probabilistic reversal learning task.

The following narrative is a response to two reflection questions:

1. What strategy did you use?
2. Did you switch strategies during the task?

Your goal is to evaluate this reflection using the Metacognitive Prompting (MP) framework.

MP Dimensions (Wang & Zhao, 2025):

You will score the reflection across the following five dimensions:

1. **Comprehension** – Did the participant understand the task rules, structure, or goal?
2. **Judgement** – Did they form a hypothesis or plan about how to act?
3. **Evaluation** – Did they reflect on whether their strategy worked, and why?
4. **Final Decision** – Did they describe a consistent rule or decision strategy they ultimately applied?
5. **Confidence** – Did they express certainty or doubt about their approach or understanding?

Scoring Rubric (used for each dimension):
Each dimension should be scored on a scale from 0 to 4, where:

- **0 = Absent** – No evidence of the dimension
- **1 = Rudimentary** – Vague or minimal expression
- **2 = Adequate** – Some clarity but not in-depth
- **3 = Good** – Clear and thoughtful expression
- **4 = Exemplary** – Sophisticated and comprehensive insight

Scoring Guidance:
Please recognize that high-quality metacognition may appear in many forms. Participants may demonstrate deep insight even if they do not explicitly state phrases like 'I evaluated' or 'I was confident'. You should reward:

- Conditional reasoning (e.g., "if…then…" statements)
- Hypothetical thinking or counterfactuals
- Thoughtful restraint and uncertainty
- Philosophical or cautious language (e.g., 'presumption of innocence')
- Rational inertia or minimal strategy change, when justified
- Reflective self-awareness (of effort, bias, or internal limits)

Short responses can still be insightful. Do not penalize indirect or abstract language if the logic and self-awareness are present.

Your Task:

For each dimension, estimate the probability that the reflection deserves a score of 0, 1, 2, 3, or 4. Respond using decimal values only (e.g., `[0.1, 0.2, 0.3, 0.2, 0.2]`). The values must:

- Be enclosed in square brackets
- Contain exactly 5 decimals
- Sum to 1.0 ($\pm$ 0.01)
- Be purely numeric (no letters)

Format your output like this:

Comprehension: [0.0, 0.1, 0.2, 0.3, 0.4]
Judgement: [0.2, 0.2, 0.2, 0.2, 0.2]
Evaluation: [0.9, 0.1, 0.0, 0.0, 0.0]
Final Decision: [0.0, 0.0, 0.0, 0.2, 0.8]
Confidence: [0.1, 0.1, 0.1, 0.3, 0.4]

Rationale: Provide a brief explanation for your probability estimates.

Scoring Tips:

- If the reflection shows deep reasoning, nuance, self-awareness, or philosophical restraint, it is okay to assign something like:
  `[0.0, 0.0, 0.0, 0.2, 0.8]`. So, you give this response a 80% probability of scoring closer to a 4 (an exemplary rating) for a particular dimension.
- If the dimension is completely absent, something like:
  `[0.9, 0.1, 0.0, 0.0, 0.0]` is acceptable. So, you give this response a 90% probability of scoring closer to a 0 (an absent rating) for a particular dimension.
- Use the full scale honestly and appropriately. Reflections with strong insight deserve strong scores (>0.7).

Supplementary Figure S2. Full metacognitive prompting script used with GPT-4. *This prompt guided GPT-4 in evaluating participant reflections on the probabilistic reversal learning task using the Metacognitive Prompting (MP) framework. Instruction included five MP dimensions (Comprehension, Judgement, Evaluation, Final Decision, Confidence), a probabilistic scoring rubric from 0 to 4, and formatting constraints for each response. The prompt also emphasizes nuance, hypothetical reasoning, and philosophical restraint as indicators of strong metacognition.*

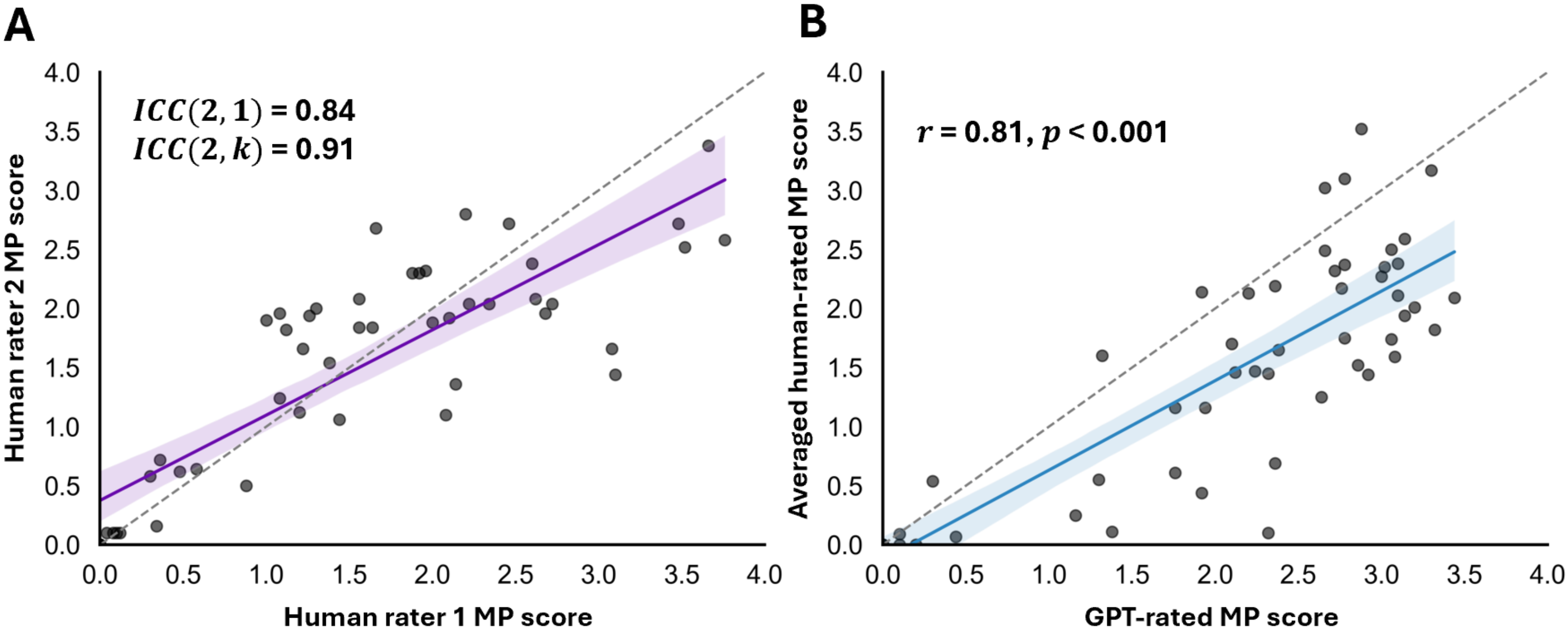

Supplementary Figure S3. Validation of GPT-derived metacognitive prompting (MP) scores against human ratings. *Scatter plots depict* ***(A)*** *agreement between two human raters and* ***(B)*** *alignment between GPT-4 and mean human MP scores. Each point represents one reflection response. The solid lines indicate best fit regressions with 95% confidence intervals (shaded regions), and dashed lines represent perfect agreement. Human-human reliability was excellent (ICC(2,1) = 0.84, ICC(2,k) = 0.91), supporting consistency between raters. GPT-4 scores strongly correlated with the averaged human ratings (r = 0.81, p < 0.001), demonstrating close correspondence between GPT's metacognitive evaluations and human judgement.*

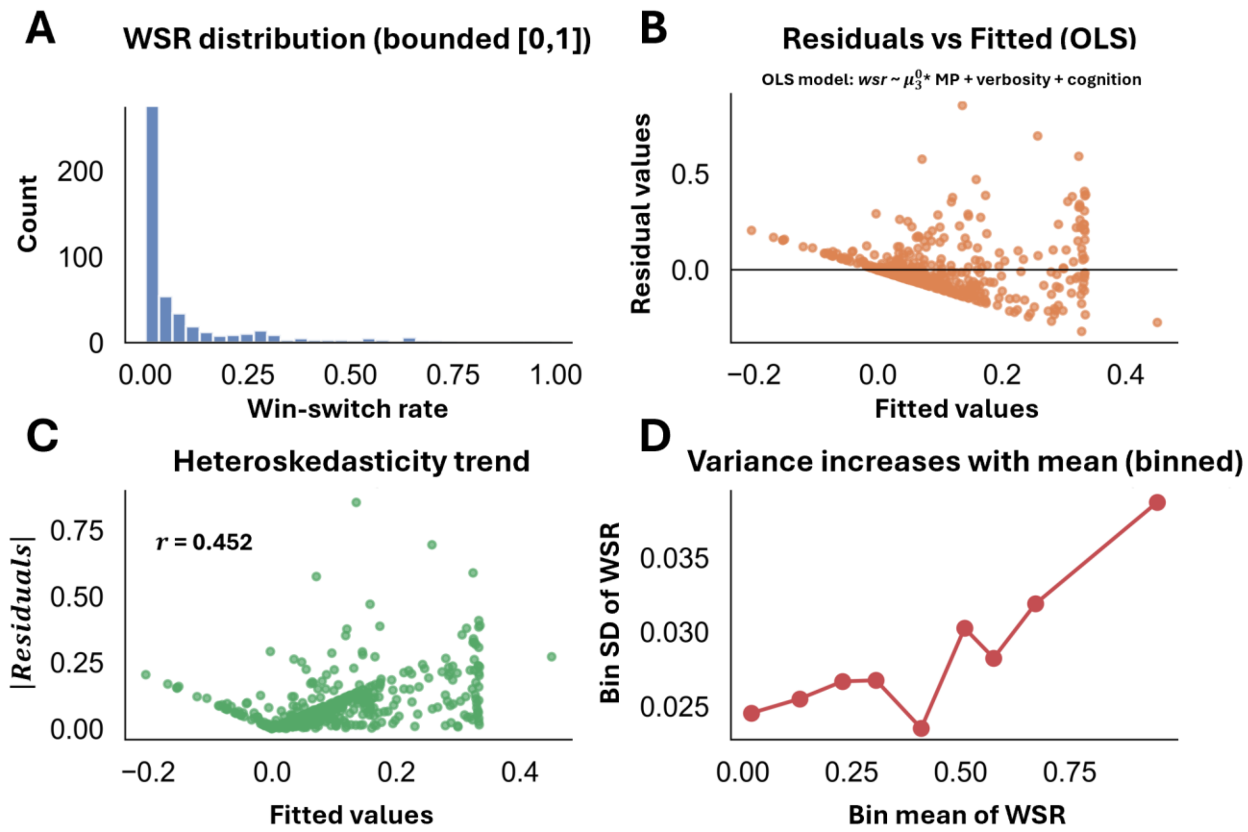

Supplementary Figure S4. Diagnostics for modeling bounded win-switch rate (WSR). *A set of plots motivating for a binomial GLM fit with a logit link.* ***(A)*** *WSR is bounded with mass near zero (min = 0, q1 = 0, median = 0.02, q3 = 0.099, max = 0.99; mean = 0.096, sd = 0.165).* ***(B)*** *Residuals fan out as fitted values increase, indicating non-constant variance.* ***(C)*** *Heteroskedasticity is evident: absolute residuals rise with the fit (r = 0.452), and a Breusch-Pagan test rejects constant variance ($\chi^2(5)$ = 44.67, p < 0.001; $F(5{,}480)$ = 9.72, p < 0.001).* ***(D)*** *Within-bin standard deviation increases with bin mean, consistent with a mean-variance coupling of proportions. Together, these diagnostics show that OLS assumptions (e.g., constant variance) do not hold for WSR and motivate using a binomial GLM (logit) that respects the [0,1] bounds.*

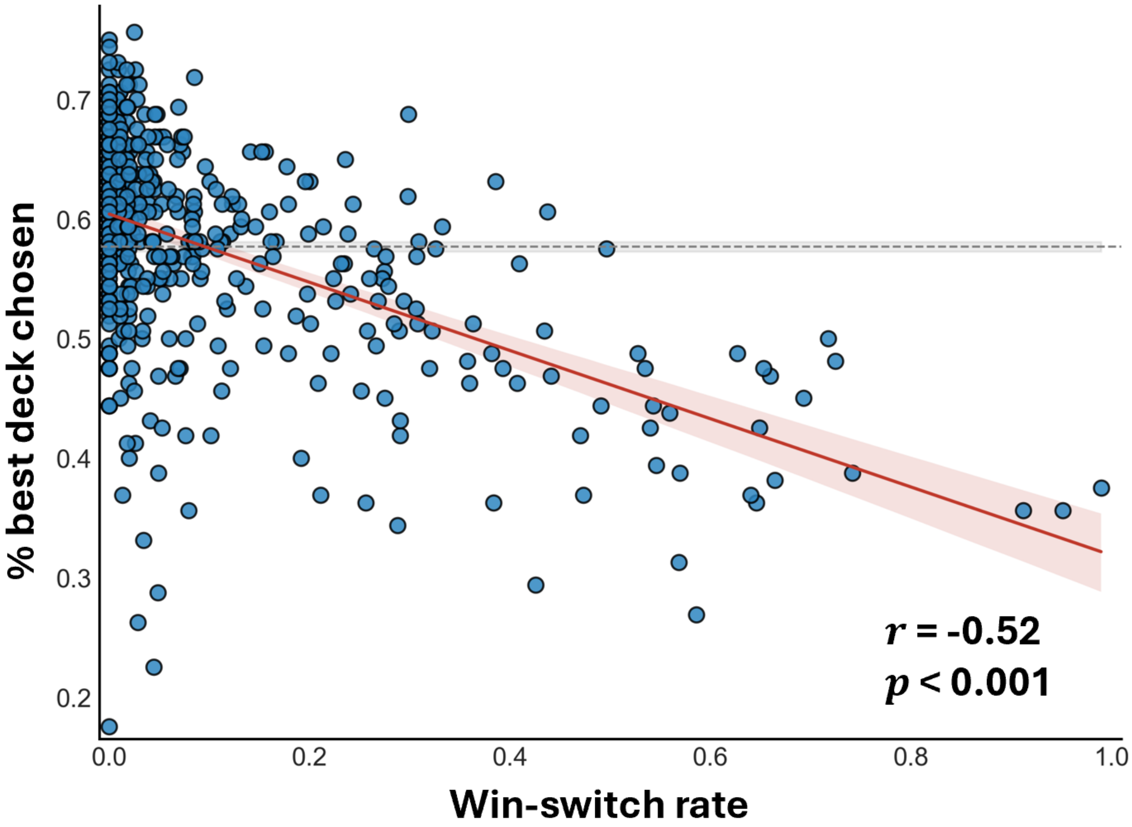

Supplementary Figure S5. Association between win switching behavior and task performance. *A scatter plot showing that higher win-switch rates predict fewer optimal choices (% best-deck chosen; r = -0.52, p < 0.001), confirming that excessive win-switching is maladaptive. The dashed horizontal line marks the sample mean proportion of optimal choices (shaded region represents the mean $\pm$ SEM across participants). The solid red line shows the best-fit regression with its 95% confidence interval (shaded region).*

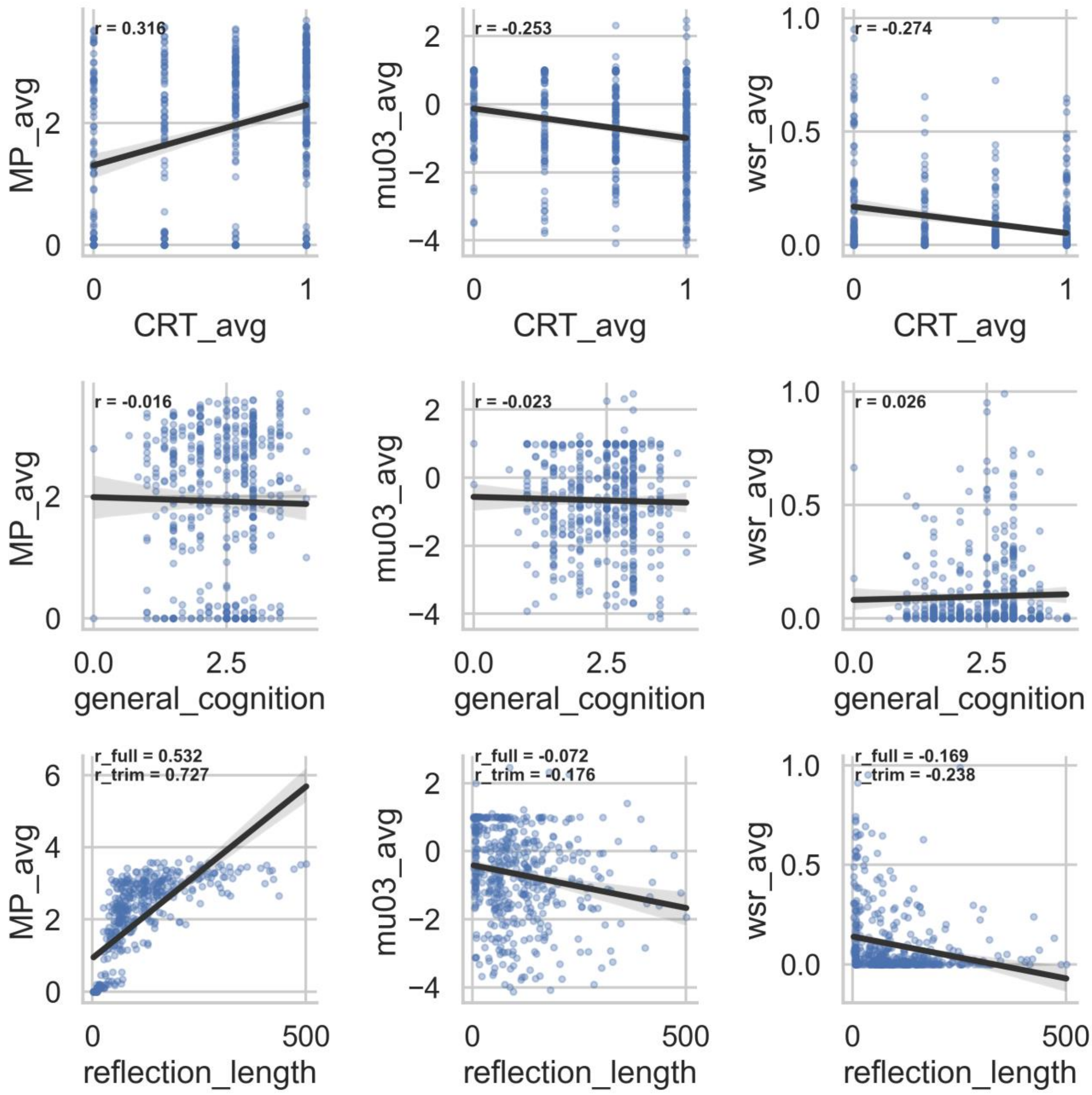

Supplementary Figure S6. Covariate-outcome correlations. *A 3x3 panel showing correlations between each covariate (CRT, general cognition, reflection length) and the three outcomes (MP, μ_3^0, WSR). For reflection length, we report both the full-sample correlation (r_full) and a trimmed sensitivity (r_trim) that excludes extreme lengths using a prespecified cutoff (max(Tukey 1.5IQR, 99.5th percentile). The 'outliers' reflect participants who wrote unusually lengthy reflections (e.g., > 2,000 characters); these are not data errors but genuine high effort responses, so we present both full and trimmed results for transparency. Correlations are small to moderate.*

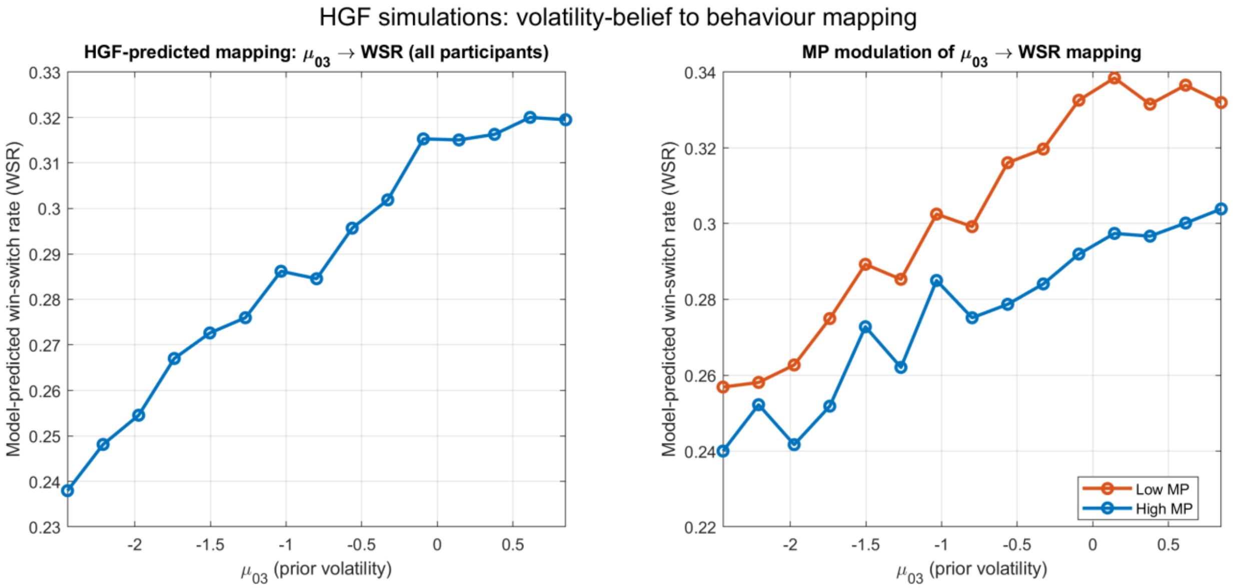

Supplementary Figure S7. Model-based simulation of metacognitive attenuation effect. *A HGF model-based demonstration that MP modulates the belief-to-behavior link. (A) Mean simulated win-switch rate (WSR), averaged across all participants, as a function of prior volatility (μ_3^0). For each participant, μ_3^0 was systematically varied across 15 values spanning the 15th-85th percentiles of the empirical μ_3^0 distribution while all other fitted HGF and softmax parameters were held fixed, and full PRL behavior was simulated to obtain model-predicted WSR. (B) The same μ_3^0 to WSR mapping shown separately for low- and high-MP participants (defined as ± 1 SD of z-scored MP). A linear fit reveals a reduced slope in the high-MP group (b = 0.0195) relative to the low-MP group (b = 0.0274) indicating MP-related differences (p = 0.005) of the belief-to-behavior mapping by metacognition. Differences in slopes were formally tested using a linear regression with an interaction term between prior volatility (μ_3^0) and metacognitive group on group-mean simulated win-switch rates. See GitHub for full simulation code.*

.

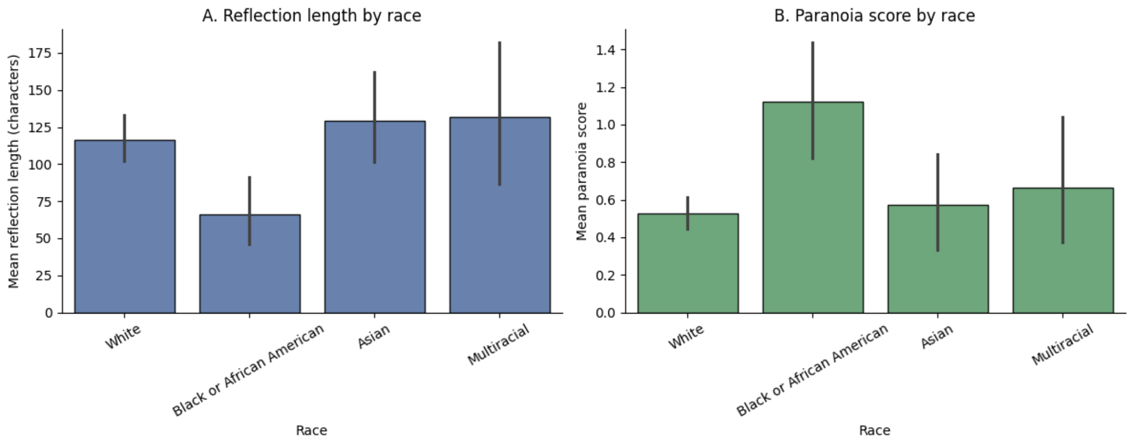

Supplementary Figure S8. Race differences in reflection length and paranoia. *(A) Mean reflection length (characters) by race. (B) Mean paranoia scores by race. Both reflection*

length ($F_{3,60.39}$ = 5.38, p = 0.002) and paranoia ($F_{3,54.26}$ = 4.49, p = 0.007) show significant variation across racial groups. For example, African American participants, on average, wrote shorter reflections and had higher paranoia scores in this sample. Because both covariates relate to MP scores, this variation helps contextualize the race-related differences observed in scoring analyses.

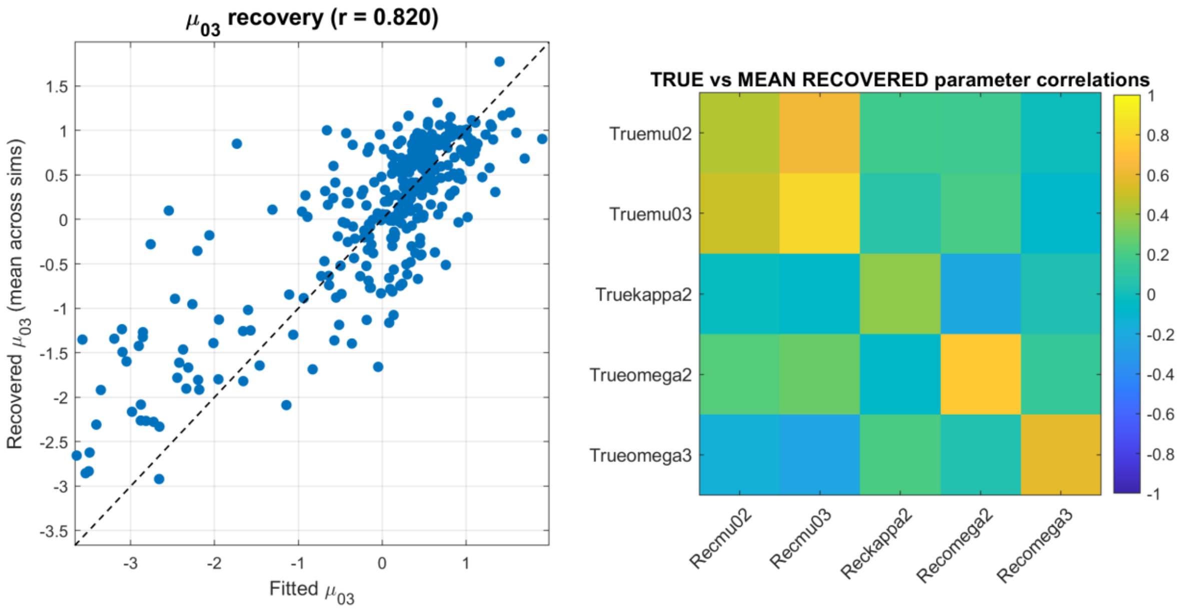

Supplementary Figure S9. Parameter recovery diagnostics. *(A)* Recovery of the volatility prior (μ_3^0) across 3 simulations (r = 0.82). Each point reflects a subject-level simulation's true (fitted) and estimated (recovered; averaged across the 3 simulations) value. *(B) Confusion matrix showing correlations between other fitted and recovered parameters. The diagonal shows positive recovery for each parameter and off-diagonal values remain modest, indicating limited trade-off between parameters. These results support the identifiability of μ_3^0 and related perceptual parameters under our HGF model configuration.*

TABLES

Supplementary Table S1. Prior mean and variance of the perceptual model. *A table summarizing the parameter configuration used for the three-level HGF model fit.*

HGF Level	Parameter	Prior mean	Prior variance
Level 3 (***Volatility learning***)	ω_3	-2	1
	ϕ_3	0.2	1
	$\mu_3(k=0)$	1	1
	$\sigma_3(k=0)$	1	1
	m_3	1	0
Level 2 (***Reward learning***)	κ_2	0.6	0.1
	ω_2	-2	1
	ϕ_2	0.4	1
	$\mu_2(k=0)$	0	1
	$\sigma_2(k=0)$	0.1	1
	m_2	0	0

Note. The prior means and variances correspond to the perceptual model parameters of the three-level HGF used for the binary multi-armed bandit task. The coupling parameter κ and the initial uncertainties σ_2 and σ_3 are estimated in *log-space*. The mean-reversion parameter ϕ is estimated in *logit-space*; μ, m, and ω are in native space. Parameters with zero prior variance were fixed during model inversion. Level 3 tracks volatility learning (environmental change rate), Level 2 tracks reward-probability learning, and Level 1 tracks the agent's experience (not estimated; selecting option 1, 2, or 3).

Dimension	Interpretation	Example (from data)
Comprehension	Refers to whether the participant demonstrates an understanding of the task structure – such as recognizing patterns, reversals or decision-relevant task contingencies.	1. *"I generally waited until I got -50 twice in a row before switching partners."*<br><br>2. *"Just tried to get the most points and not stick with people generating negative points."*
Judgement	Captures the participant's ability to generate and assess hypotheses under uncertainty. This includes early interpretations or beliefs about patterns or partner behavior.	1. *"At first I tried to count how many iterations a good card remained good."*<br><br>2. *"Tried to stick with the person that had 100 points most of the time."*
Evaluation	Assesses whether the participant reflected on why their strategies worked or failed. It captures feedback-based reasoning and causal explanation of outcomes.	1. *"Switching did not work as well near the end. I had to stick with one even after a -50 because all others were worse."*<br><br>2. *"I noticed one student failed once after a long streak, so I gave them another chance."*
Final Decision	Indicates whether the participant converged on a consistent behavioral rule or strategy by the end of the task. It reflects policy formation or strategy adherence.	1. *"I would usually stick with a student until failing an assignment, and then look for another student who performs better."*<br><br>2. *"I just clicked a good card until it became bad, then switched."*
Confidence	Evaluates expressions of certainty or uncertainty about choices or task understanding. Includes hedges, qualifiers or confident assertions about performance.	1. *"I tried to take some risks but mostly stayed with my safer guesses."*<br><br>2. *"I didn't. Just tapping about and seeing what was what"*

Supplementary Table S2. Five Dimensions of Metacognition. A summary table of the five core dimensions of metacognition, interpreted with some real reflections after completing the PRL.

Supplementary Table S3. Scoring Rubric*. Range for each metacognition dimension.*

Score	*Category*	*Meaning*
0	Absent	No evidence of the dimension
1	Rudimentary	Vague or minimal expression
2	Adequate	Some clarity but no in-depth
3	Good	Clear and thoughtful expression
4	Exemplary	Sophisticated and comprehensive insight

Supplementary Table S4. Covariates distribution. *CRT, general cognition, and reflection length distribution.*

Variable	*Mean*	*SD*	*Min*	*Q1*	*Median*	*Q3*	*Max*
CRT	0.62	0.39	0	0.33	0.67	1	1
General cognition	2.43	0.72	0	2	2.5	3	4
Reflection length	110.53	141.17	3	36.25	81	147.75	2313

Supplementary Table S5. Covariates-only binomial GLM. *Effects of covariates on WSR.*

Predictors	**OR**	**AME**	**SE**	***p***
Reflection (length)	0.99	-0.0005	0.0002	0.01
Cognition (general)	1.07	0.0056	0.0187	0.76

Covariates-only model: *win-switch rate ~ reflection_length + general_cognition*

Supplementary Table S6. Demographic distribution. *Gender and race distribution.*

Variable	Group	N	Percent
Gender	Female	193	39.71
	Male	289	59.47
	Other	4	0.82
Race	White	367	75.51
	Black	59	12.14
	Asian	30	6.17
	Multirracial	20	4.12
	Other	1	0.21
